# Caveolin-3 protects diabetic hearts from acute myocardial infarction/reperfusion injury through β2AR, cAMP/PKA, and BDNF/TrkB signaling pathways

**DOI:** 10.18632/aging.103469

**Published:** 2020-07-21

**Authors:** Jiaji Gong, Fan Zhou, Simin Xie, Xin Wang, Junmei Xu, Feng Xiao

**Affiliations:** 1 Department of Anesthesiology, The Second Xiangya Hospital, Central South University, Changsha 410011, China

**Keywords:** DM (diabetes mellitus), acute myocardial infarction injury, cAMP/PKA signaling, BDNF/TrkB signaling

## Abstract

Diabetes mellitus (DM) might increase the incidence and mortality of cardiac failure after acute myocardial infarction (AMI) in patients. We attempted to investigate whether Caveolin-3 showed beneficial effects in DM patient post-MI injury through the cAMP/PKA and BDNF/TrkB signaling pathways. The activity of ADRB2 and cAMP/PKA signaling were impaired in nondiabetic ischemia-reperfusion (I/R) group compared with the sham and DM groups and were more impaired in diabetic I/R group than in the I/R group. In H9C2 cells, high-glucose (HG) stimulation further enhanced H/R injury by promoting cell apoptosis, inhibiting cell viability, and suppressing TrkB and Akt signaling; in contrast, the ADRB2 agonist isoprenaline (ISO) significantly attenuated the above-described effects of HG stimulation. Caveolin-3 overexpression promoted the localization of ADRB2 on the membrane of the HG-stimulated H9C2 cells, subsequently inhibiting apoptosis and promoting cell viability. Under HG stimulation, Caveolin-3 overexpression enhanced the activity of the cAMP/PKA and BDNF/TrkB signaling pathways, whereas ADRB2 silencing reversed the effects of Caveolin-3 overexpression. In conclusion, ADRB2 agonist promoted the activity of the BDNF/TrkB and cAMP/PKA signaling pathways, mitigating the HG-aggravated H/R injuries in H9C2 cells. Caveolin-3 exerts a protective effect on diabetic hearts against I/R damage through the β2AR, cAMP/PKA, and BDNF/TrkB signaling pathways.

## INTRODUCTION

Diabetes mellitus (DM) is regarded as a metabolic disease. Deficiencies in insulin secretion, hyperglycemia, and hyperlipidemia are the major characteristic features of DM. Recently, the incidence of diabetes has gradually increased, and the major cause of the death in diabetic patients is cardiovascular complications [1]. Epidemiological studies have found that the incidence and mortality of cardiac failure in diabetic patients after acute myocardial infarction (AMI) were significantly higher than those in nondiabetic patients [2]. This phenomenon might be related to the sensitivity of diabetic myocardium to myocardial ischemic injury; however, the mechanism for failure in post-AMI repair in DM patients is unclear.

Increasing evidence has shown that neurotrophic factors, including nerve growth factor, can promote myocardial neovascularization and improve myocardial blood flow and heart function to exert beneficial effects on the heart after myocardial infarction [3]. Brain-derived neurotrophic factor (BDNF) is a secreted protein in the neurotrophic factor family, that has a neuroprotective effect against oxygen-glucose deprivation (OGD) [4]. The specific binding of BDNF to tropomyosin-related kinase receptor B (TrKB) could further modulate downstream intracellular signaling pathways, therefore affecting the nervous system development and function [5]. In hearts with MI, BDNF/TrkB relieved myocardial ischemic injury and suppressed myocardial cell apoptosis via the regulation of transient receptor potential canonical (TRPC) 3/6 channels, thus suggesting a new underlying therapy for MI [6]. It has been reported that cyclic adenosine monophosphate (cAMP) could rapidly promote the localization on the cytomembrane and phosphorylation of the TrkB receptor, subsequently enhancing the transduction of the BDNF/TrkB signaling pathway [7]; cAMP/ protein kinase A (PKA)/ cAMP-response element binding protein (CREB) could promote the expression of TrkB [8]. Thus, cAMP/PKA signaling pathway is an essential upstream signaling that might modulate the BDNF/TrkB signaling activity, therefore affecting post-MI impairment in the diabetic heart.

However, this essential upstream signaling is impaired in the diabetic heart post-MI, mainly due to inhibition of the β-adrenergic pathway that produces cAMP [9]. Under hyperglycemic, hyperlipidemic, or low insulin conditions, myocardial beta-adrenergic receptor (β2AR) expression is reduced, and cardiovascular dysfunction occurs [10]. An online gene chip profile (GSE12639) indicated that a total of 753 genes are differentially-expressed in rats with streptozotocin (STZ)-induced diabetic MI, compared with nondiabetic rats with MI, and 18 genes a (log_2_|FC| > 0.56, *p* < 0.05), including γ-aminobutyric acid receptor, cholinergic receptor, urotensin receptor, endothelin receptor, adrenergic receptor β2 (ADRB2), melanocortin 5 receptor and oxytocin receptor were significantly different. ADRB2 was significantly downregulated (log_2_FC = -0.73, *p* <0.05) [11]. Moreover, we performed Kyoto Encyclopedia of Genes and Genomes (KEGG) annotation analysis and found that the differentially-expressed genes in the diabetic rats with MI were significantly concentrated in the neuroactive ligand-receptor pathway (rno04080: neuroactive ligand-receptor interaction). These previous studies suggest that, in diabetic rats, the response of cardiomyocytes to neuroactive factors in the cellular microenvironment is dysregulated. The β2AR pathway is impaired in the diabetic environment and may affect the downstream cAMP/PKA pathway in the myocardium, which in turn affects BDNF/TrkB pathway activity and impedes the repair of myocardial ischemia-reperfusion (I/R) injury.

Caveolin-3 is a protein known to protect against myocardial I/R injury. Caveolin-3 regulates cardiomyocyte β2AR (ADRB2) localization on the membrane and mediates downstream cAMP signaling pathways [12–14]. In ventricular myocytes from normal hearts, the constitutive modulation via Caveolin-3 is limited to T-tubules. in cardiac failure, constitutive modulation via Caveolin-3 is lost [14]. However, whether Caveolin-3 promotes the BDNF/TrkB downstream signaling pathway through the β2-adrenergic receptor/cAMP/PKA is unknown. Herein, we established an STZ-induced DM model in myocardial I/R rats and an H/R (hypoxia/reoxygenation) injury model in H9C2 cells under HG (high glucose)/NG (normal glucose) condition; examined the changes in the β2AR, cAMP/PKA, and BDNF/TrkB signaling pathways; and investigated whether Caveolin-3 improves the repair of H/R injury and I/R injury *in vitro* and *in vivo*, respectively, through the β2AR, cAMP/PKA, and BDNF/TrkB signaling pathways. In summary, we provide a solid experimental basis for a novel mechanism of Caveolin-3 protecting against myocardial I/R injury in DM hearts.

## RESULTS

### Myocardial I/R injury in rats with diabetes mellitus (DM)

**Figure 1:**
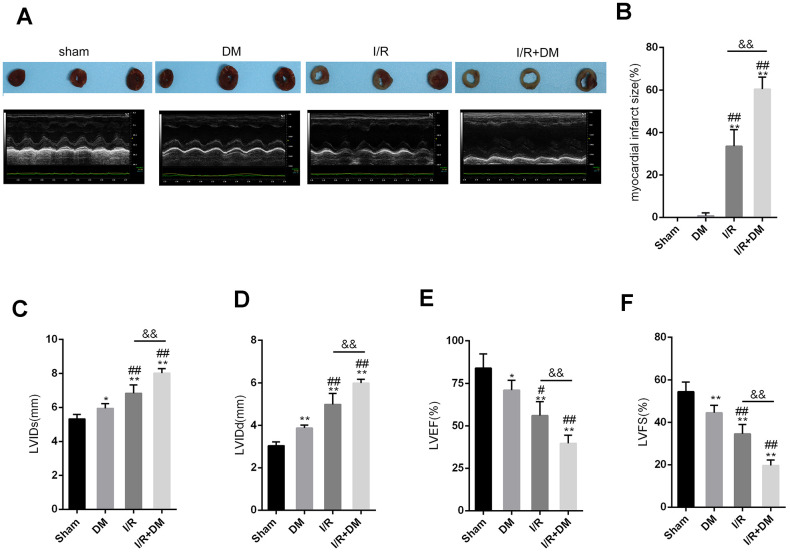
**Myocardial ischemia reperfusion (I/R) injury model in diabetes mellitus (DM) rats SD rats were subjected to DM induction and then received coronary artery ligation to induce myocardial infarction (MI).** Rats were randomly assigned to three groups: sham surgery group, DM group, I/R group, and I/R + DM group. (**A**) Representative photos of myocardial infarct size. Four weeks after I/R operation, rats were then examined for (**A**, **C**, **D**, **E**, and **F**) LVIDs, LVIDd, LVEF, and LVFS by echocardiography; (**B**) myocardial infarct size. N=5. **P<0.01, compared to sham group; #*P*<0.05, ##*P*<0.01, compared to DM group; && *P*<0.01, compared to I/R group.

Rats were subjected or not subjected to induction of DM and evaluated for blood sugar and body weight once every 10 days for 60 days. As shown in Supplementary Figure 1A–1B, STZ injection significantly induced increases in the rat blood sugar and body weight in a time-dependent manner. Next, rats with DM received coronary artery ligation to induce I/R injury and then underwent the echocardiographic and pathological examinations. All rats were randomly assigned to 4 groups: the sham surgery group, DM group, I/R group and I/R + DM group. Representative photos of myocardial infarct area showed that a larger infarct area was observed within the single I/R group than the control groups and the largest area was observed in the I/R + DM group (Figure 1A). DM induction significantly increased the infarct size in the I/R + DM group, compared with the in single I/R group (Figure 1B). Next, left ventricular internal diameter end-systolic (LVIDs), Left ventricular end diastolic diameter (LVIDd), Left Ventricular Ejection Fraction (LVEF), and Left ventricular fraction shortening (LVFS) were determined by echocardiography. The echocardiography results showed that the LVIDs and LVIDd were significantly increased, whereas the LVEF and LVFS were reduced in DM, I/R and I/R + DM groups compared to the sham group (Figure 1C–1F). Moreover, LVIDs and LVIDd were more increased, whereas the LVEF and LVFS were reduced in the I/R + DM group, compared to I/R group, indicating that DM induction further damaged cardiac function.

### The cAMP/PKA and β-adrenergic signaling pathways upstream of TrkB are dysregulated in the I/R and diabetic I/R groups

**Figure 2:**
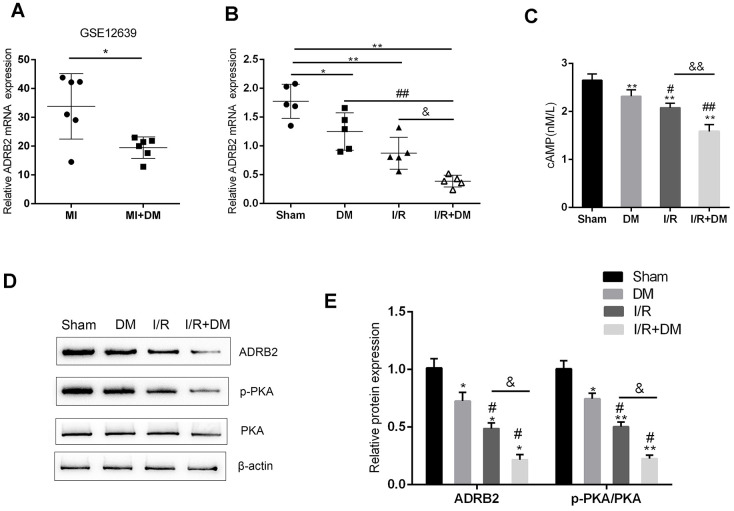
**cAMP/PKA and β-adrenergic signaling pathways upstream of TrkB are dysregulated in I/R and diabetic I/R group**. (**A**) ADRB2 mRNA expression in MI and MI + DM rats based on online microarray profile (GSE12639). (**B**) ADRB2 mRNA expression in rat hearts (n=5) from Sham, DM, I/R, or I/R + DM group determined by real-time PCR. (**C**) The concentration of cAMP in rat hearts from Sham, DM, I/R, or I/R +DM group determined by ELISA. (**D**, **E**) The protein levels of ADRB2, p-PKA, and PKA in rat hearts from Sham, DM. I/R, or I/R + DM group determined by Immunoblotting, n=3. **P<0.01, compared to sham group; #*P*<0.05, ##*P*<0.01, compared to DM group; && *P*<0.01, compared to I/R group.

As we have mentioned, the cAMP/PKA pathway is impaired in DM, mainly because the upstream β-adrenergic pathway that produces cAMP is inhibited [9]. According to an online microarray profile (GSE12639), the mRNA expression of ADRB2 (the gene that encodes adrenergic receptor β2) was significantly downregulated in the I/R + DM rats, compared to the I/R rats (Figure 2A). In the present study, PCR-based analysis also revealed that ADRB2 mRNA expression was strongly downregulated in the rat hearts (n=5) from the DM, I/R and I/R + DM groups compared to that from the sham surgery group (Figure 2B) and was much lower in the I/R + DM group than in the DM and I/R group (Figure 2B). Consistent with a previous study, the cAMP concentration in the rat hearts from the DM, I/R and I/R + DM groups showed to be remarkably reduced than that from the sham surgery group (Figure 2C), and was more reduced in the I/R + DM group than in the single I/R group (Figure 2C). Further confirming of the impaired cAMP/PKA signaling pathway, the ADRB2 protein and PKA phosphorylation levels were strongly decreased in the DM, I/R and I/R + DM groups compared with the sham group (Figure 2D), more reduced in the I/R + DM group than the single I/R group (Figure 2D–2E). In summary, the cAMP/PKA signaling pathway and the upstream β-adrenergic pathway were both impaired in the DM, I/R and I/R + DM groups, more impaired in the I/R + DM group than the other groups.

### An ADRB2 agonist activates cAMP/PKA signaling and the recovery of H9C2 cells from H/R injury under HG stimulation

**Figure 3:**
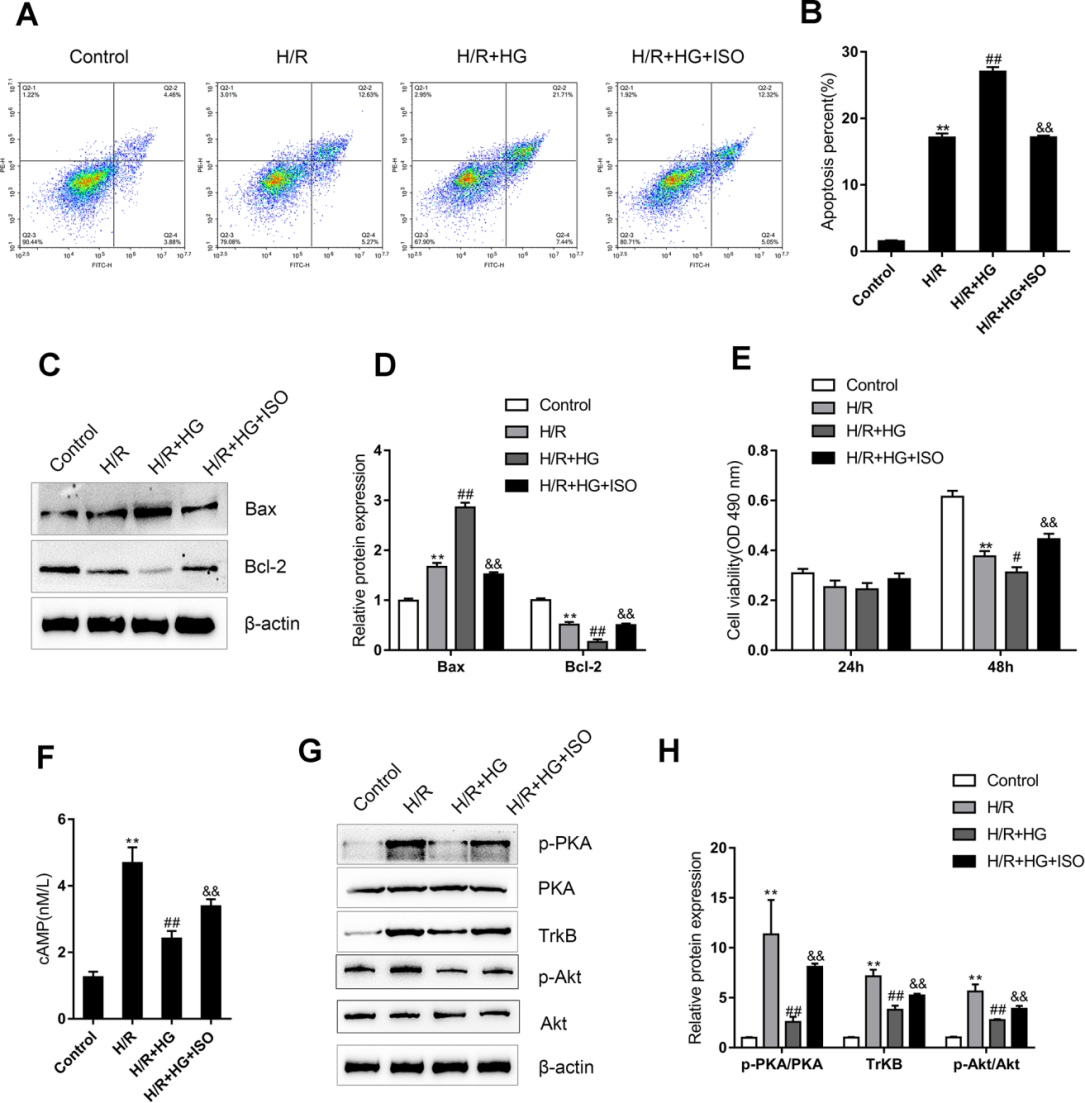
**An ADRB2 agonist activates cAMP/PKA signaling and the recovery of H9C2 cells from H/R injury under high-glucose (HG) stimulation.** H9C2 cells were subjected to H/R injury with or without HG stimulation and ADRB2 agonist ISO treatment and examined for (**A**, **B**) the cell apoptosis by Flow cytometry (n=3); (**C**, **D**) the protein levels of apoptotic Bax/Bcl-2 signaling factors Bax and Bcl-2 by immunoblotting (n=3); (**E**) the cell viability by MTT assay (n=5); (**F**) the cAMP concentrations (n=3); (**G**, **H**) the protein levels of p-PKA, PKA, TrkB, p-Akt, and Akt by immunoblotting, (n=3).**P<0.01, compared to control group; #*P*<0.05, ##*P*<0.01, compared to H/R group; &&*P*<0.01, compared to H/R + HG group.

Since the β-adrenergic pathway and ADRB2 expression are impaired in diabetic I/R rats, we next, constructed cell models to further investigate the underlying molecular mechanism. H9C2 cells were subjected to H/R injury in the absence or presence of HG stimulation and the ADRB2 agonist ISO and examined for related indexes. Cell apoptosis was significantly promoted by both single H/R injury and the H/R + HG combination and showed a greater increase with the H/R + HG combination (Figure 3A–3B); the administration of the ADRB2 agonist significantly reduced cell apoptosis (Figure 3A–3B). Consistently, single H/R injury and H/R + HG combination significantly increased Bax while decreasing the Bcl-2 protein levels, and the H/R + HG combination had a stronger effect on these two proteins (Figure 3C–3D); the administration of the ADRB2 agonist significantly decreased Bax while increasing the Bcl-2 protein levels (Figure 3C–3D). Regarding cell viability, H/R injury and the H/R + HG combination significantly inhibited cell viability, and the H/R + HG combination more strongly inhibited cell viability (Figure 3E); Administration of theADRB2 agonist reversed the suppressive effects of the H/R + HG combination on cell viability (Figure 3E).

Regarding the cAMP/PKA signaling pathway, H/R injury induced sharp stress-responsive increases in the cAMP concentrations, p-PKA/PKA ratios, TrkB protein levels, and p-Akt/Akt ratios while the H/R + HG combination significantly attenuated these increases (Figure 3F–3G); the administration of the ADRB2 agonist reversed the inhibitory effects of the H/R + HG combination on the cAMP concentrations, p-PKA/PKA ratios, TrkB protein levels, and p-Akt/Akt ratios (Figure 3F–3G). These data suggest that the ADRB2 agonist activates cAMP/PKA signaling to improve H/R injury under HG stimulation in H9C2 cells.

### Caveolin-3 promotes the localization of β2AR on the cytomembrane of H9C2 cells

**Figure 4:**
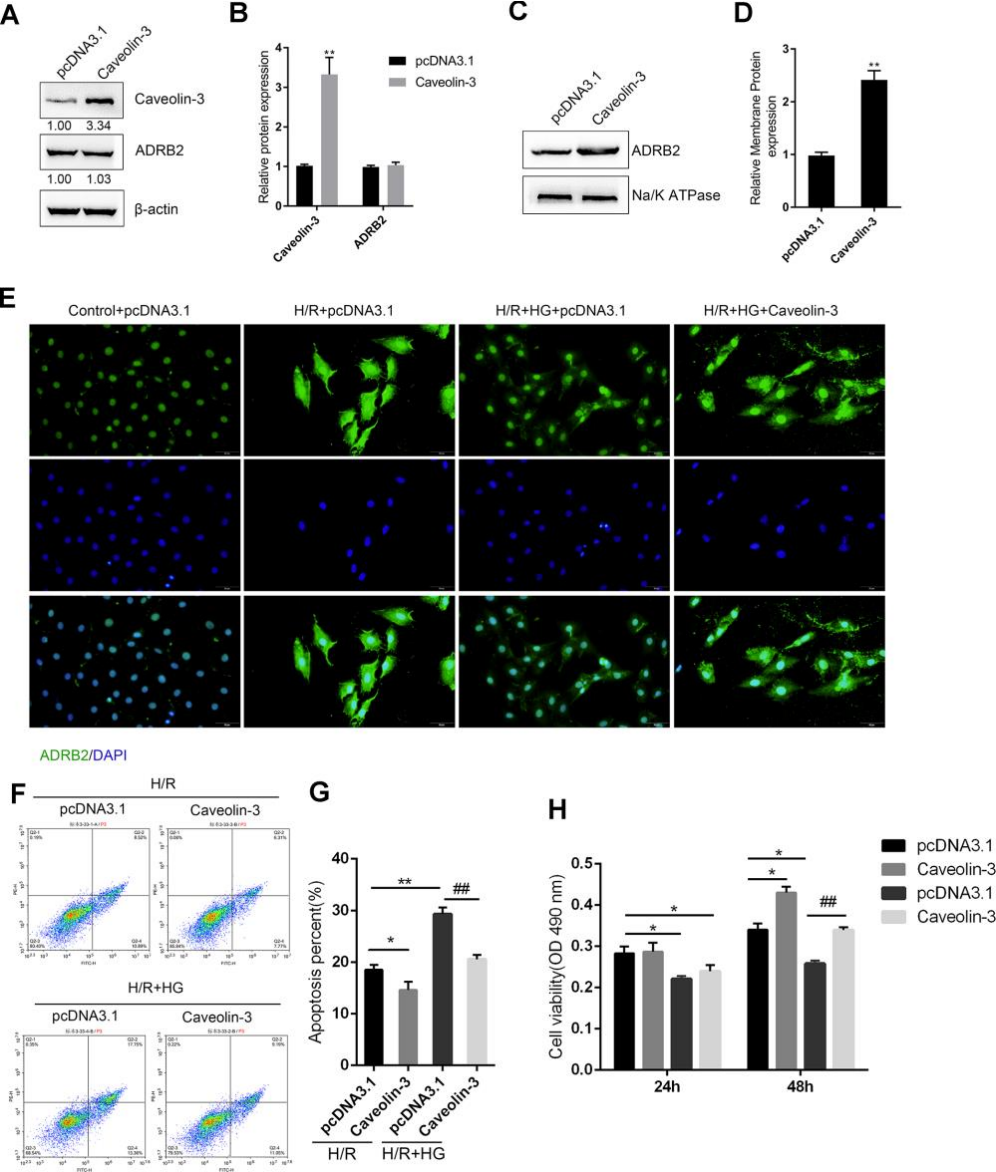
**Caveolin-3 promotes the localization of β2AR onto the cytomembrane of H9C2 cells.** (**A**, **B**) H9C2 cells were transfected with pcDNA3.1/Caveolin-3 to conduct Caveolin-3 overexpression. The total protein levels of Caveolin-3 and ADRB2 in Caveolin-3-transfected cells were determined using immunoblotting, (n=3). (**C**, **D**) The membrane protein levels of ADRB2 in Caveolin-3-transfected cells were determined using immunoblotting, (n=3). (**E**) The content and distribution of ADRB2 protein in the control, H/R, H/R + HG, and H/R + HG + Caveolin-3 groups determined by IF staining, (n=3). (**F**–**H**) H9C2 cells were transfected with pcDNA3.1/Caveolin-3 under H/R or HG stimulation and examined for cell apoptosis by Flow cytometry (n=3) and cell viability by MTT assay (n=5). *P<0.05, **P<0.01 compared to pcDNA3.1+H/R group; ##*P*<0.01, compared to pcDNA3.1+H/R group.

Since Caveolin-3 has been reported to promote the localization of β2AR on the cytomembrane and mediate the downstream cAMP signaling [12–14], we next, investigated whether Caveolin-3 could protect H9C2 cells against H/R injury upon HG stimulation by affecting the localization of β2AR on the cytomembrane. H9C2 cells were transfected with pcDNA3.1/Caveolin-3 to induce Caveolin-3 overexpression, as confirmed by immunoblotting (Figure 4A–4B); in the Caveolin-3-overexpressing H9C2 cells, there was no significant alteration in total ADRB2 protein (Figure 4A–4B). However, Caveolin-3 overexpression indeed significantly increased the membrane protein levels of ADRB2 (Figure 4C–4D). The content and distribution of the ADRB2 protein were then determined by IF staining in H9C2 cells of the control+pcDNA3.1 group, H/R+pcDNA3.1 group, H/R + HG+ pcDNA3.1 group, and H/R + HG + Caveolin-3 group. H/R injury induced a sharp stress-responsive increase in the ADRB2 protein content while the H/R + HG combination inhibited this increase; Caveolin-3 overexpression increased the fluorescence intensity indicating ADRB2 on the membrane (Figure 4E). In addition, Caveolin-3 overexpression significantly inhibited cell apoptosis but promoted cell viability under H/R or H/R+HG stimulation (Figure 4F–4H). These data suggest that Caveolin-3 overexpression promotes the localization of β2AR on the cytomembrane of H9C2 cells under H/R injury and HG stimulation, thus rescuing H9C2 cell viability.

### Caveolin-3 enhances BDNF/TrkB signaling activity through ADRB2 and cAMP

**Figure 5:**
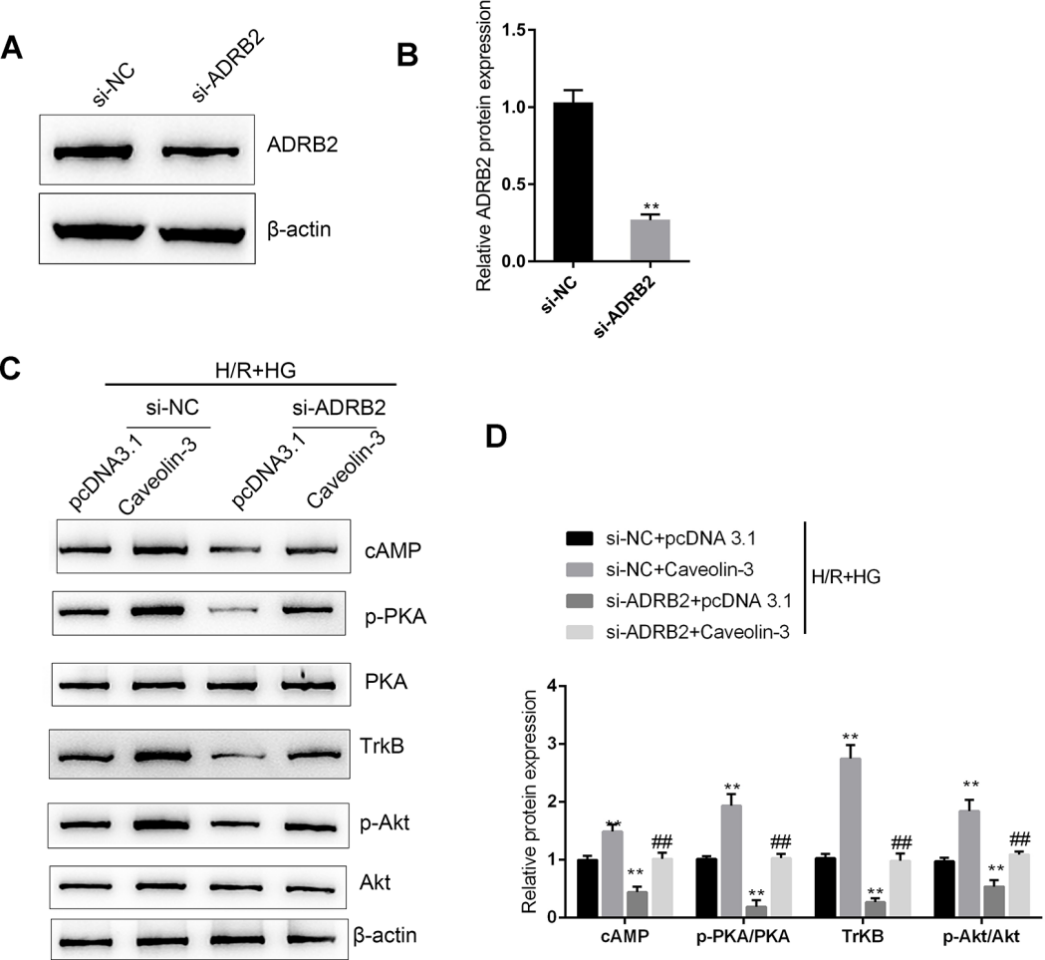
**Caveolin-3 enhances the activity of BDNF/TrkB signaling through ADRB2 and cAMP.** (**A**, **B**) ADRB2 silencing was conducted in H9C2 cells by transfection of si-ADRB2, as confirmed by immunoblotting. Next, under H/R+HG stimulation, H9C2 cells were co-transfected with pcDNA3.1/Caveolin-3 and si-ADRB2 and examined for (**C**, **D**) cAMP, p-PKA, PKA, TrkB, p-Akt, and Akt proteins. N=3. **P<0.01 compared to si-NC group or pcDNA3.1+si-NC group; ##*P*<0.01, compared to pcDNA3.1+si-ADRB2 group.

Next, we investigated whether Caveolin-3 exerts its effects by modulating BDNF/TrkB signaling via ADRB2 and cAMP. We transfected si-ADRB2 to conduct ADRB2 silencing in H9C2 cells and performed Immunoblotting to verify the transfection efficiency (Figure 5A–5B). Next, we cotransfected H9C2 cells with pcDNA3.1/Caveolin-3 and si-ADRB2, and then evaluated the cAMP, p-PKA, PKA, TrkB, p-Akt, and Akt protein levels under H/R+HG stimulation. In H9C2 cells under H/R+HG stimulation, Caveolin-3 overexpression was strongly upregulated, whereas ADRB2 silencing downregulated the cAMP and TrkB proteins, and PKA and Akt phosphorylation (Figure 5C–5D); the effects of Caveolin-3 overexpression on β-adrenergic, cAMP, and BDNF/TrkB signaling were partially reversed by ADRB2 silencing (Figure 5C–5D). These data suggest that Caveolin-3 enhances the activity of BDNF/TrkB signaling through the ADRB2 and cAMP signaling pathways.

## DISCUSSION

Here, we established an I/R model and diabetic I/R model in rats and found that I/R injury in the I/R + DM group was more severe than that in the I/R group. Online microarray profile and PCR-based analysis revealed that the activity of ADRB2 (β2AR) and cAMP/PKA signaling were impaired in the single I/R group compared with the sham group and were more impaired in I/R + DM group than in I/R group. In H9C2 cells, HG stimulation further enhanced H/R injury by promoting cell apoptosis, inhibiting cell viability, and suppressing TrkB and Akt signaling; in contrast, ADRB2 agonist ISO significantly attenuated the effects of HG stimulation on the H/R injured H9C2 cells. Caveolin-3 overexpression promoted the localization of ADRB2 to the membrane in HG-stimulated H9C2 cells, subsequently inhibiting the apoptosis and promoting the cell viability of H9C2 cells. Under H/R+HG stimulation, Caveolin-3 overexpression enhanced the activity of the cAMP/PKA and BDNF/TrkB signaling pathways, whereas ADRB2 silencing reversed the effects of Caveolin-3 overexpression.

Increasing epidemiological and clinical evidence has revealed that the hearts of diabetic patients show increased susceptibility to I/R damage [15–17]. Reportedly, DM can not only aggravate MIR damage, but also weaken the protection of numerous therapeutic drugs [18, 19]. Hyperglycemia is regarded as an independent risk factor that can worsen heart function, cellular survival, and tissue damage after myocardial I/R [20]. Herein, an I/R model was generated in rats with STZ-induced DM. This model could be used in pathophysiological studies of type 2 diabetes [21–23]. Along with the increases in blood glucose and body weight, heart function was more impaired in I/R + DM group than in the I/R group, as manifested by the increased infarct size, increased LVIDs and LVIDd, and decreased LVEF and LVFS. These findings were consistent with the previous study [24, 25].

The protective effects of the BDNF/TrkB and cAMP/PKA signaling pathways against I/R injury have been reported. Hang et al. [6] demonstrated that BDNF/TrkB regulated TRPC3/6 channels to relieve myocardial I/R injury and to suppress the apoptosis of myocardial cells. These researchers also indicated that BDNF mitigated the proapoptotic effect of miR-195 on rat cardiomyocytes, while its scavenger TrkB-Fc could counteract this effect [26]. The cAMP/PKA/CREB signaling pathway exerts a critical effect on neurogenesis [27]. CREB phosphorylated by PKA can target the CRE element of the promoter and, thereby promote BDNF transcription [28]. Moreover, upstream β2AR signaling, which has been regarded as the main source of cAMP, is also inhibited in the DM myocardium, leading to impaired cAMP/PKA signaling [9]. Herein, we found that the cAMP content was dramatically reduced in the hearts from the I/R + DM group compared to that from the single I/R group; moreover, the protein levels of ADRB2 and p-PKA were strongly reduced in diabetic I/R hearts compared to I/R hearts, indicating that the β2AR and cAMP/PKA signaling pathways were both impaired in the hearts from the I/R group and the I/R + DM group and more impaired in the hearts from the I/R + DM group. Further *in vitro* experiments revealed that HG stimulation further enhanced H/R injuries in H9C2 cells, whereas the ADRB2 agonist ISO improved the H/R injuries in H9C2 cells under HG stimulation, as shown by the inhibited cell apoptosis and enhanced cell viability. In addition, the activity of the BDNF/TrkB and cAMP/PKA signaling pathways was partially reversed by ISO administration in the H/R-injured H9C2 cells under HG stimulation, suggesting that the ADRB2 agonist could activate the BDNF/TrkB and cAMP/PKA signaling pathways, therefore mitigating the HG-aggravated H/R injuries in H9C2 cells.

Caveolin-3 is considered a master type of caveolin family protein in cardiomyocytes that forms caveolae [29] and acts as a platform on the cell membrane to regulate signaling pathways through signaling molecules anchored in caveolins. Previous studies have demonstrated that both caveolae and Caveolin-3 play an essential role in the inducible effects of exendin-4 on heart protection against I/R damage [30]. In the present study, Caveolin-3 overexpression significantly promoted the localization of β2AR to the membranes, therefore promoting the H/R-injured H9C2 cell viability and inhibiting cell apoptosis under HG stimulation. More importantly, the activity of the cAMP/PKA and BDNF/TrkB signaling pathways was enhanced by Caveolin-3 overexpression, whereas inhibited by ADRB2 silencing; the effects of Caveolin-3 overexpression were partially reversed by ADRB2 silencing, indicating that caveolin-3 protects H9C2 cells from H/R injury under HG stimulation via the β2AR, cAMP/PKA, and BDNF/TrkB signaling pathways.

## CONCLUSIONS

An ADRB2 agonist promotes the activity of the BDNF/TrkB and cAMP/PKA signaling pathways, therefore mitigating the HG-aggravated H/R injuries in H9C2 cells. Caveolin-3 exerts a protective effect on diabetic hearts against I/R damage through the β2AR, cAMP/PKA, and BDNF/TrkB signaling pathways.

## MATERIALS AND METHODS

### Induction of DM in rats

Male Sprague-Dawley rats (SD rats) aged 2-3 months (with an average weight of 300 g) were obtained from the SLAC experimental animal center (Changsha, China). DM was induced in SD rats via a single peritoneal injection of STZ at a dose of 40 mg/kg [31–33] following a protocol approved by the Institutional Animal Care and Use Committee at The Second Xiangya Hospital. The animal experiments were approved by our institutional review board. When the blood glucose level reached 13 mM (234 mg/dL), the rats were fed a high-fat diet consisting of 21.2% protein, 12% fat, 15% sucrose and 1% cholesterol for 2 months. The control rats were fed normal rodent food consisting of 20% protein and 4.5% fat. Then, the rats in both the STZ-diabetic and normal control groups underwent sham or coronary artery ligation to induce myocardial I/R. Briefly, Rats were anaesthetized intraperitoneally with Nembutal (40 mg/kg). Myocardial ischemia was performed by a temporary tightening (30 min) of the silk ligature around the left main coronary artery as previously described [34]. Reperfusion was achieved by releasing the tension applying to the ligature (I/R groups). Sham group rats underwent all the same surgical procedures without coronary artery ligation.

### Blood glucose determined by the Accutrend Plus test strips and meter

Blood samples were collected from the tail vein for the determination of blood glucose levels prior to induction of MI and at the end of study using Accutrend Plus test strips and meters (Roche, Basel, Switzerland) as described previously [31–33] to further verify DM.

### Left ventricular (LV) function in rats determined by echocardiography

Transthoracic echocardiographic images of the hearts from the sham, DM, I/R and I/R + DM groups of rats were obtained at 4 weeks post-MI or sham using an ultrahigh-resolution ultrasound scanner (Vevo 2100; VisualSonics) under isoflurane anesthesia. LVIDs, LVIDd, LVEF and LVFS were recorded as described previously [31–33]. The evaluation of the results was performed by sonographers unaware of the study design.

### RT-PCR

Total RNA from cardiac tissues was extracted using TRIzol reagent (Invitrogen;), following the manufacturer’s procedures. Complementary DNA was synthesized from extracted RNA using the Prime Script^®^ RT Reagent kit with gDNA Eraser (Invitrogen) in accordance with the recommended protocol. RT-PCR was performed using SYBR^®^Premix Ex Taq^™^ II (Invitrogen). ABI7500 real-time PCR detection system (Applied Biosystems, USA) was used for detection. β-actin expression levels were used as endogenous control. Finally, the data were processed using the 2^-ΔΔCt^ methods.

### Immunoblotting

Tissues or cells were lysed in RIPA lysis buffer (Beyotime, China). For membrane protein isolation, cells were lysed by membrane and Cytosol Protein Extraction Kit, according to the manufacture’s instruction (Beyotime). Proteins extracted from tissues or cells were separated by SDS-PAGE, transferred to a polyvinylidene difluoride membrane (Millipore, Burlington, MA, USA), and then incubated with the primary antibody followed by anti-rabbit or anti-mouse IgG conjugated with horseradish peroxidase (Abcam, Cambridge, MA, USA). The primary antibodies used were as follows: anti-TrkB (ab18987), anti-Akt (ab32505), anti-p-Akt (ab81283), anti-Bax, anti-Bcl-2anti-ADRB2 (ab182136), anti-PKA (ADI-KAS-PK017-F; Enzo, Hong Kong, China), anti-p-PKA (ab75991), and anti-Caveolin-3 (ab2912). β-actin (6008-1-Ig, ProteinTech, Rosemont, IL, USA) was used as an endogenous control for total protein. Na/K ATPase (sc-514614, Santa Cruz, USA) was used as an endogenous control for membrane protein. All antibodies were obtained from Abcam unless otherwise stated.

### Intracellular cAMP concentration determined by ELISA

The intracellular cAMP concentration was determined by a rat cAMP ELISA kit (DEIA2964, Creative Diagnostics, Shirley, NY, USA). Target cells were plated in a 24-well plate at a cell density of 5 × 10^5^ cells/well. After treatment and washing with ice-cold PBS, cells were lysed with RIPA buffer (Beyotime). The supernatant was obtained by a centrifugation and examined for the concentration of cAMP using cAMP ELISA kit.

### Cell line and cell culture

H9C2 cells, rat embryonic myoblasts, were obtained from ATCC (Manassas, VA, USA) and cultured in DMEM supplemented with 10% FBS, 100 units/ml penicillin, and 100 mg/ml streptomycin at 37°C in 5% CO_2_.

### Cell transfection

The Caveolin-3 overexpression vector, empty pcDNA3.1 vector, si-NC and si-AADRB2 were purchased from Genetop Medical Tech.co. ltd (Changsha, China). 1 μg/ml vector or 20 nM siNRA were transfected into H9C2 cells using Lipofectamine 2000 (Invitrogen). Forty-eight hours after transfection, cells were harvested for further experiments. The primers for plasmid construction and sequence for interference were listed in Supplementary Table 1.

### H/R model in H9C2 cells under HG/NG conditions

H9C2 cells were placed in a hypoxic vessel filled with the mixture of 94% N_2_, 5% CO_2_ and 1% O_2_ for 5 min, subjected to hypoxia for 12 h at 37°C and reoxygenated for 12 h by exposing the cells to a cell incubator. The cells were stimulated with NG (5 mmol/L glucose) or HG (30 mmol/L glucose) in the presence or absence of the ADRB2 agonist isoproterenol (ISO, 20μM) during reoxygenation.

### Cell viability determination by MTT assay

A modified MTT assay was used to evaluate cell viability following the previously described methods [35]. DMSO was added after the supernatant discarded to dissolve the formazan. The OD values were measured at 490 nm. The viability of the nontreated cells (control) was defined as 100%, and the viability of the cells from all other groups was calculated separately from that of the control group.

### Analysis of cell apoptosis examined by flow cytometry

Cell apoptosis was determined using an Annexin V-FITC apoptosis detection kit (Keygen, China) following methods described previously [36]. Propidium iodide (PI) was used for nuclear staining. The excitation wavelength (Ex) was 488 nm and the emission wavelength (Em) was 530 nm.

### ADRB2 protein determined by immunofluorescence (IF) analysis

ADRB2 protein content and distribution were analyzed by performing IF staining using anti-ADRB2. The secondary antibody FITC-conjugated donkey anti-rabbit IgG (1:500) was obtained from Abcam. The nucleus was stained with DAPI. The green fluorescence represents the ADRB2 protein and blue fluorescence represents the nucleus.

### Data analysis and statistics

All data from at least three independent experiments were analyzed using SPSS 13.0 software (SPSS, Chicago, IL, USA) and are presented as the mean ± SD. The differences between groups were analyzed by one-way ANOVA (normal distribution), and multiple comparisons were performed by the Bonferroni correction. Differences with *P* < 0.05 were considered statistically significant.

### Ethics approval

The protocol in the study was approved by the Institutional Animal Care and Use Committee at The Second Xiangya Hospital. The animal experiments were approved by our institutional review board.

### Availability of data and material

All data generated or analyzed during this study are included in this published article. Further details are available on request.

### Consent for publication

Consent for publication was obtained from the participants.

## Supplementary Material

Supplementary Figure 1

Supplementary Table 1
