# Supplementary material for: Caveolin-3 protects diabetic hearts from acute myocardial infarction/reperfusion injury through β2AR, cAMP/PKA, and BDNF/TrkB signaling pathways
Source: Aging (Albany NY). 2020 Jul 21;12(14):14300–13. doi: 10.18632/aging.103469 (PMC7425465; doi:10.18632/aging.103469)
Supplement: Supplementary Figure 1 [file aging-12-103469-s002..pdf]

SUPPLEMENTARY MATERIALS

Supplementary Figure

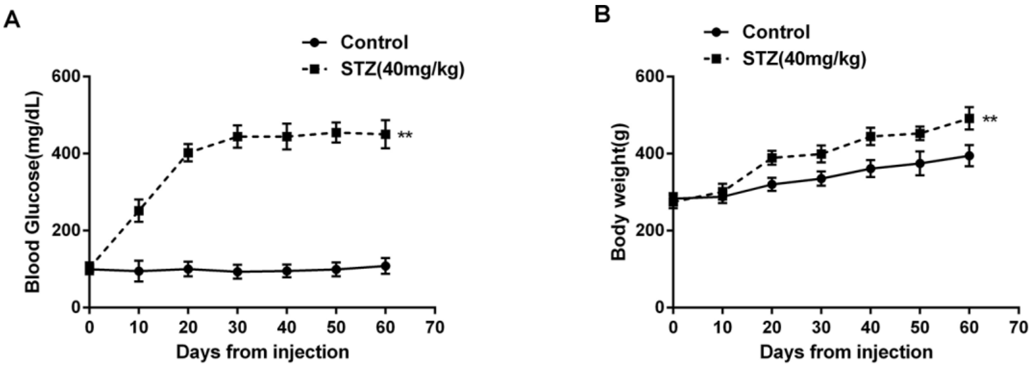

Supplementary Figure 1. Construction of DM model in SD rats. The blood glucose (**A**) and the body weight (**B**) were determined once 10 days for 60 days. N=10. $^{**}P<0.01$.
