# Supplementary material for: Caveolin-3 protects diabetic hearts from acute myocardial infarction/reperfusion injury through β2AR, cAMP/PKA, and BDNF/TrkB signaling pathways
Source: Aging (Albany NY). 2020 Jul 21;12(14):14300–13. doi: 10.18632/aging.103469 (PMC7425465; doi:10.18632/aging.103469)
Supplement: Supplementary Table 1 [file aging-12-103469-s001..pdf]

SUPPLEMENTARY MATERIALS

Supplementary Table

Supplementary Table 1. The primer sequences.

Name	Forward	Reverse
ADRB2-qPCR	GCCACGACATCACTCAGGAACG	AGTCCAGAACTCGCACCAGAAATTG
β-actin-qPCR	GGTGAACAGAGACCCCAAGAACATC	GCCCAGATGTGGCAGAAGGAGATA
Caveolin-3 overexpression vector (pcDNA3.1)	CCCAAGCTTATGATGACCGAAGAGCACACAGAT	CCGGAATTCTTAGCCTTCCCTTCGCAGCAC
Si-NC	UUCUCCGAACGUGUCACGUTT	ACGUGACACGUUCGGAGAATT
Si-ADRB2	GGUCAAGUAUUAAGGAUAATT	UUAUCCUUAAUACUUGACCTT
